# Aerial Trajectories and Meteorological Drivers of Transboundary *Loxostege sticticalis* Migration Across Northern China and Mongolia, 2022

**DOI:** 10.3390/insects17020218

**Published:** 2026-02-19

**Authors:** Xing-Yue Pu, Yi-Yang Zhang, Hai-Bin Gu, Rui Zhong, Gui-Jun Wan, Fa-Jun Chen, Qiu-Lin Wu

**Affiliations:** 1 School of Ecology and Applied Meteorology, Nanjing University of Information Science & Technology, Nanjing 210044, China; 202312080018@nuist.edu.cn (X.-Y.P.); guhbin@hotmail.com (H.-B.G.); 202113020085@nuist.edu.cn (R.Z.); 2 Jiangsu Provincial University Key Laboratory of Agricultural and Ecological Meteorology, Nanjing University of Information Science & Technology, Nanjing 210044, China; 3 National Agro-Tech Extension and Service Center, Beijing 100125, China; zhangyiyang@agri.gov.cn; 4 College of Plant Protection, Nanjing Agricultural University, Nanjing 210095, China; guijunwan@njau.edu.cn (G.-J.W.); fajunchen@njau.edu.cn (F.-J.C.)

**Keywords:** *Loxostege sticticalis*, spatiotemporal dynamics, source population, Mongolia, NCCV

## Abstract

Clarifying migration pathways and the source area–destination relationships of the domestic and foreign beet webworm *Loxostege sticticalis* (Linnaeus) populations, as well as understanding the meteorological mechanisms shaping these processes, is pivotal for remote, accurate, and location-specific pest early warning and forecasting. Based on light trap data from northern China and field survey data from Mongolia in 2022, we simulated the migration trajectories, source regions, and primary landing areas of *L. sticticalis* by using the HYSPLIT model and analyzed the synoptic systems, processes and conditions during its migration. The results indicate the frequent exchange of *L. sticticalis* populations between China and Mongolia in 2022. The *L. sticticalis* migrants initiating their flights from Mongolia primarily undertook a southeastward migration pathway, supplemented by eastward ‘cyclonic’ and southwestward paths. The main landing areas were located in North China and Northeast China, with migration events potentially extending to the Shandong, Heilongjiang, and Xinjiang provinces. Populations originating from North China exhibited a capacity for migrating into Northeast China and Mongolia through 1–5 consecutive nights of flight. During this period, the Northeast China Cold Vortex (NCCV) and the Mongolian Cyclone alternately regulated the synoptic circulation pattern governing the migration of *L. sticticalis*. The spatiotemporal distributions and intensities of these systems were key determinants of the transboundary migration routes and distances of *L. sticticalis*. The NCCV dominated, and the precipitation and downdrafts it induced were crucial for the massive landing of *L. sticticalis* in northern China.

## Simple Summary

The beet webworm *Loxostege sticticalis* (Linnaeus) is a major migratory pest that frequently occurs in the temperate regions of northern China. To understand the source area–destination relationships and the meteorological mechanisms between northern China and Mongolia *Loxostege sticticalis* (Linnaeus) populations during the peak period, this study examined the migration pathways of this pest in 2022 using light trap data from northern China and field surveys in Mongolia. Results show significant population exchanges between two countries, with a primary southeastward migration route from Mongolia, along with eastward and southwestward routes. Major landing areas were identified in Northern and Northeast China. Populations from North China can migrate into Northeast China and Mongolia after 1–5 successive nights of flight. The Northeast China Cold Vortex (NCCV) and Mongolian Cyclones remarkably influence the synoptic patterns that govern migration, with their spatiotemporal characteristics critical to determining transboundary routes and distances of *L. sticticalis* migrants.

## 1. Introduction

The beet webworm *Loxostege sticticalis* (Linnaeus) belongs to the Pyralidae family in the order Lepidoptera. It is characterized by its migratory ability, destructive nature, periodic outbreaks, and omnivorous diet [1], with its range mainly spanning the narrow strip of Eurasia between 36° N and 55° N, including countries such as China and Mongolia [2,3]. *L. sticticalis* infests more than 50 host plant species across 21 families, mainly damaging crops such as soybean and sunflower, and poses a serious threat to agriculture and animal husbandry in China’s Three North Regions [4]. During severe outbreak years, it can inflict yield losses ranging from approximately 60% to a staggering 100% [5,6]. *L. sticticalis* ranks third among Category 1 crop pests, as announced by the Ministry of Agriculture and Rural Affairs, following only *Spodoptera frugiperda* and *Locusta migratoria*.

China has been expanding planting acreage, boosting the planting of soybean and other host crops, and reducing the planting of maize as part of its national goals, such as the Rural Vitalization Strategic Plan. This may help *L. sticticalis* populations spread [7]. Since the founding of the People’s Republic of China, *L. sticticalis* has undergone three major outbreak cycles: 1955–1961, 1978–1984, and 1996–2009 [8]. After the third cycle, infestations remained at low levels from 2010 to 2016 [9]. In 2018, however, high densities of first-generation larvae were detected in the border regions of Inner Mongolia, Heilongjiang, and Jilin [10], followed by a marked increase in overwintering adults in 2019 [11]. The population regained strength in 2020, signaling the initiation of a new severe outbreak cycle [12]. Additionally, global warming has intensified the northward expansion of this pest’s overwintering range in North China and Northeast China, and promoted the westward migration of its second- and third-generation adults to high-altitude areas [13]. The distribution of *L. sticticalis* adults in North China is widespread and severe, with the activity regions of pests from the overwintering generation to the first generation advancing northward [14]. In Northeast China, the peak trapping period for overwintering-generation adults has come earlier, with large moth populations, prolonged duration, and multiple migration peaks [15]. Contrary to the conventional view in the 1980s, Chen et al. [6] proposed that North China serves as a source of migrant *L. sticticalis* populations for Northeast China, but not the primary source. The pest populations primarily originate from local overwintering groups and those from neighboring countries such as Mongolia. Part of them come from the border areas between China and Mongolia, China and Russia, or the tri-border region of the three countries [16,17]. *L. sticticalis* has long been widely distributed in Russia, Mongolia, and other regions [18]. In 2008, although the population of first-generation larvae in China was small, a massive influx of first-generation adults migrating from Russia and Mongolia resulted in a severe outbreak of second-generation larvae [19]. Moreover, Mongolia shares a long border with China, and contains similar vegetation types on which *L. sticticalis* primarily feeds [20].

Long-distance migration is initiated under favorable atmospheric conditions, which can be significantly disrupted by cyclonic systems. For instance, the cut-off low observed in Northeast China, designated as the Northeast China Cold Vortex (NCCV), is a persistent and relatively stable atmospheric system present between 35 and 60° N and 110–145° E, regularly occurring from May to August each year [21,22]. This spatial and temporal range coincides with the migration period and geographic distribution of *L. sticticalis*. Due to their weak flight ability, *L. sticticalis* typically migrates with favorable winds [23,24]. It can be greatly influenced by near-surface winds, low-level jets, and, particularly, severe air convection at the migration altitude [25,26]. This pest is forced to land when the temperature falls below 15 °C or the cumulative precipitation across 12 h reaches 0.1 mm [27,28]. The NCCV, typically accompanied by squall lines, storms, and heavy rainfall [29,30], gives rise to prolonged rain, low temperatures, and abrupt severe convective weather in Northeast China and Inner Mongolia [31,32]. Previous studies, such as those by Sun et al. [15] and Chen [33], have found that the migration direction of *L. sticticalis* is closely related to the near-surface winds influenced by the NCCV. Additionally, Chen [33] suggests that the precipitation and downdrafts, which lead to its mass landings, may be induced by the NCCV. Moreover, low temperature and rainfall can also have a significant effect on the growth and production of crops [34,35], thereby indirectly affecting how often outbreaks of *L. sticticalis* occur and how much harm they cause. The Mongolian Cyclone, which occurs in regions between 42.5° N–55° N and 85° E–120° E [36], overlaps with the distribution areas of *L. sticticalis* in China and Mongolia [16], and is often associated with strong winds and frost [37]. While it is most prominent in spring, it can also be observed year-round [38]. This cyclonic system significantly influences near-surface wind fields, and its strong winds can affect insect migration [39].

In 2022, according to the report from the National Agro-Tech Extension and Service Center, the occurrence area of *L. sticticalis* was approximately 2.74 × 10^5^ hm^2^, which is close to the average occurrence area between 2020 and 2024. This makes 2022 suitable for analyzing the migration of *L. sticticalis* during a typical occurrence year. Moreover, previous investigations regarding this migratory pest have mainly been conducted within China, and the spatiotemporal transboundary connectivity between domestic and foreign migratory populations remains to be fully elucidated. Therefore, the limited yet valuable field survey data of *L. sticticalis* moths recorded in Mongolia in 2022 serves as a strong data basis for clarifying the overseas origins of this pest in China. In this study, we used the Hybrid Single-Particle Lagrangian Integrated Trajectory (HYSPLIT; https://www.ready.noaa.gov/HYSPLIT.php accessed on 11 December 2024) model, in conjunction with ArcGIS 10.7, to perform the migration pathways of *L. sticticalis* populations in China and Mongolia in 2022. Additionally, Python 3.10.18 was utilized to analyze the meteorological conditions influencing *L. sticticalis* migration to elucidate its migration processes and landing mechanisms, thereby providing a theoretical foundation for the prediction, early warning, and management of this pest.

## 2. Materials and Methods

### 2.1. Pest Occurrence Data

**Figure 1:**
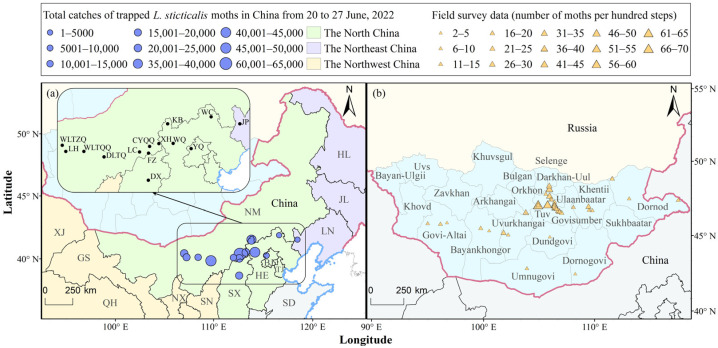
Light-trap catches of overwintering *L. sticticalis* during the peak period of light-trap catches in northern China (**a**) and field survey data from June to August in Mongolia (**b**) (2022). HL—Heilongjiang; JL—Jilin; LN—Liaoning; NM—Inner Mongolia; BJ—Beijing; TJ—Tianjin; HE—Hebei; SD—Shandong; SX—Shanxi; SN—Shaanxi; NX—Ningxia; GS—Gansu; QH—Qinghai; XJ—Xinjiang. WLTZQ—Urad Middle Banner; LH—Linhe District; WLTQQ—Urad Front Banner; DLTQ—Dalad Banner; CYQQ—Chahar Right Front Banner; XH—Xinghe County; FZ—Fengzhen City; LC—Liangcheng County; DX—Dai County; WC—Weichang County; KB—Kangbao County; WQ—Wanquan District; YQ—Yanqing District; JP—Jianping County.

The occurrence data of *L. sticticalis* moth, including searchlight trap records, black-light trap records, and female moth ovary dissection results from northern China in late June 2022, as well as field survey data (number of moths per hundred steps) from Mongolia between June and August 2022, were provided by the National Agro-Tech Extension and Service Center. The monitoring data are shown in Figure 1, and Table 1 and Table 2.

The searchlight and black-light traps used in this study comply with the guidelines outlined in the Construction Specification for Observation Fields for Crop Diseases and Insect Pests (NY/T3698-2020) [40]. In China, searchlight traps are set up in open areas, such as elevated platforms, to ensure no interference from tall buildings or strong light sources. These traps include a support frame, time controller, insect collector, funnel, and a 1000 W metal halide lamp, which remains operational even during rainy conditions. The lamp emits light at a wavelength of approximately 600 nm and is mounted at the focal point of a parabolic reflector, directing light into a large column that extends 500 to 1000 m upward [41,42], effectively attracting migrating insects. The lights are turned on at sunset and off at sunrise, with insects collected the following morning for sorting, counting. On peak days, ovarian development stages were examined.

Black-light traps (known as ground-based light traps) are placed in areas free from tall buildings or powerful light sources within 100 m. A 20 W blacklight bulb (wavelength 365 nm) is used, with the lamp installed 1.5 m above the ground. The lights are turned on at sunset and off at sunrise, with insects being sorted, counted, and examined the following morning.

The field survey methodology selects representative areas such as farmland, grasslands, and forestlands for investigation. For each area, a random point is selected, and the investigator walks 100 steps at a normal pace while visually counting the number of moths disturbed by investigator’s movement. This process is repeated multiple times, and the average count is recorded.

### 2.2. Meteorological Data

The study utilized the ERA5 dataset (ECMWF Fifth Generation Reanalysis; https://cds.climate.copernicus.eu/ accessed on 16 December 2024), a global climate reanalysis product provided by the European Centre for Medium-Range Weather Forecasts (ECMWF), to extract meteorological data from 28 May to 13 August 2022, with a spatial resolution of 0.25° × 0.25° and a temporal resolution of 1 h. For this study, we selected the spatial domain of 85–140° E, 30–60° N—covering Northeast China, Northwest China, North China, and Mongolia—as the specific research area. Given that *L. sticticalis* migration typically occurs at night, we conducted meteorological analyses using Python, focusing on meteorological elements/conditions from 18:00 to 06:00 Beijing Time (UTC+8) on the following day. In the study area, the average elevations of monitoring stations are about 1000 m in China and 1287 m in Mongolia, with stations below 800 m accounting for less than 12.5%. We decided to examine predominant factors including the wind field (u and v components), vertical velocity, geopotential height, temperature at the 850 hPa level, and surface precipitation [1]. We utilized the geopotential height at 850 hPa to identify cyclones in Mongolia [43], while 500 hPa geopotential height and temperature were the criteria for defining the NCCV [22].

### 2.3. Simulating Migration Paths Using the HYSPLIT Model

Cooperatively produced by the National Oceanic and Atmospheric Administration’s (NOAA) Air Resources Laboratory and the Australian Bureau of Meteorology, the HYSPLIT model is popularly used not only to trace the spread of air masses in order to understand their paths and other intricate processes, including transit, transformation, diffusion, and deposition. *L. sticticalis* migrants are typically transported by airflows, with an average flight speed of 2.5–4.6 km/h [23]. Their migration direction generally aligns with the wind direction [24], although the specific orientation has not yet been reported. Therefore, in this work, given the relatively weak flight capacity of the *L. sticticalis*, we utilized the HYSPLIT model to simulate the migration paths of both domestic and foreign *L. sticticalis* migrating populations, thus identifying potential source areas or landing regions [44,45,46]. Moreover, the HYSPLIT model has been widely adopted to simulate the migration paths of various windborne insect species, such as fall armyworm (*Spodoptera frugiperda*) [47], boll weevils (*Anthonomus grandis)* [48], corn earworm (*Helicoverpa zea)* [49], and other migrating insects [50,51,52]. In particular, this trajectory model for migratory moths was validated by Wu et al., [53].

Referring to the Rules for Forecast Technology of the Meadow Moth [Loxostege sticticalis (Linnaeus)] in Agricultural Areas (DB 22/T 2477-2016) [54], which states that the insect source captured by searchlight traps are primarily considered as migratory individuals, we assessed whether it might have individuals with potential for migration. The identification of *L. sticticalis* migration events was based on the synchronous detection of both searchlight and black-light traps. Specifically, a monitoring site could suggest having experienced a migration event when *L. sticticalis* moths were simultaneously monitored by both types of traps. In the present study, during the peak period of light-trap catches, migratory populations where the proportion of female moths with ovaries at stage III–IV exceeds 65% were considered likely immigrants, while those below this ratio were emigrants. Although the application of the criteria for characterizing migratory population—specifically distinguishing between immigrants or emigrants in *L. sticticalis*—remains limited, similar methodologies have been widely applied in studies of other species, such as the oriental armyworm (*Mythimna separata*) [55], rice leafroller (*Cnaphalocrocis medinalis*) [56], brown rice planthopper (*Nilaparvata lugens*) [57], and fall armyworm (*Spodoptera frugiperda*) [58]. The migration pathways and main landing areas of *L. sticticalis* emigrants were determined by conducting forward trajectory analysis during the peak period of light-trap catches, while backward trajectories were employed to analyze the potential source regions of immigrants. At LH (Inner Mongolia), ovarian dissection data were obtained from the black-light trapping samples but not for the searchlight trap. We therefore selected the corresponding data on dates when *L. sticticalis* moths were monitored by both light traps to support its migration analysis. For DX (Shanxi), where ovarian dissection data were not available for 2022, population status in late June 2022 was inferred from historical monitoring records for the same period in 2020–2021 [12,14]. Field evidence in 2020 and 2021 indicated the presence of local populations in late June [14]; In particular, forward trajectory analysis were performed for DX to investigate potential emigration trajectory of *L. sticticalis* in late June 2020 [12].

According to Frolov et al. [18], the overwintering generation of this pest in Mongolia begins to appear from mid- to late May. To fully explore the migration trajectories of *L. sticticalis* moths from Mongolia, it is assumed that adults documented there were potential migrants of various ages. Historical records and laboratory findings suggest that the preoviposition period for migratory populations is 5.13 days [59]. It implies that the observed migratory *L. sticticalis* moths in Mongolia may be 1–5 days old. In addition, previous studies indicate that *L. sticticalis* moths typically migrate for 1–5 successive nights [12]. For instance, *L. sticticalis* moths documented in the field on 13 June 2022 were estimated to have emerged as early as 9 June 2022, and were expected to takeoff and engage in their migration during 9 to 17 June 2022. Thus, we defined the ‘Migration Time Window’ (MTW) as date(s) the 4 d before and 4 d after the actual monitoring date, and the estimated MTW for different sites are specified in Table 2.

*L. sticticalis* typically takes off around sunset, migrates during the night, and lands at dawn, with a flight duration of approximately 10 h, and migration altitudes between 300 and 500 m above ground level (AGL), with the core insect layer centered around 400 m AGL. Radar data indicate a scarcity of individuals over 700 m AGL, with moths typically flying for 1–5 nights [24,44]. In this study, for each day during the peak period of light-trap catches, we simulated the migratory routes of *L. sticticalis* based on specific biological traits and computational parameters: (1) Migration simulations were conducted for 1 to 5 consecutive nights, with 20:00 Beijing Time (UTC+8) serving as the starting point for forward calculation and endpoint for backward calculation, and vice versa for 06:00 the next day. (2) The endpoint of each night’s trajectory served as the starting point for the following night, and flight duration for each night could last a maximum of 10 h. (3) Flight altitudes were set at intervals of 100 m between 200 and 700 m. (4) During each night’s flight, it was assumed that *L. sticticalis* could potentially land at any full hour. Utilizing historical occurrence data and records of *L. sticticalis* in China (Table S1) and [60], we collected overwintering/breeding regions at the prefecture-level city resolution that could potentially serve as valid source/sink regions for *L. sticticalis* migration. Thus, endpoints derived from backward/forward trajectory analysis located in both large bodies of water and invalid source/sink regions were excluded. Finally, valid endpoints were statistically analyzed using ArcGIS 10.7. The probability was calculated as the ratio of valid endpoints within the target provinces to the total valid endpoints. To enhance the clarity of the trajectory outputs, we included only representative trajectories that illustrate the main migration directions. In selecting these trajectories, we prioritized stations with higher catches numbers of *L. sticticalis* moths and significant latitudinal and longitudinal differences among them. The complete dataset of trajectories is available in the Supplementary Materials (Figures S1 and S2). The standard map of China plotted in this study (Map Review No.: GS (2024) 0650) was downloaded from the National Platform for Common Geospatial Information Services (https://www.tianditu.gov.cn/).

### 2.4. Meteorological Data Analysis and Visualization

All analyses were performed using Python 3.10.18. Data processing and visualization relied on the following key packages: ‘xarray’ for reading multidimensional meteorological datasets; ‘pandas’ for data preprocessing and date formatting; and ‘numpy’ for numerical computations, including the extraction of station-specific meteorological variables such as air temperature, wind speed, wind direction, vertical velocity at 850 hPa, and surface precipitation. Precipitation intensity was classified according to the Chinese national standard Grade of Precipitation (GB/T 28592-2012) [61].

For figure generation, ‘matplotlib’ was used to create plots, standardize colormaps, and fine-tune typographic details. The spatial distributions of precipitation, air temperature, geopotential height at 850 hPa and 500 hPa were visualized using the ‘ax.contourf’. The 850 hPa wind field was represented using the ‘ax.quiver’ function to display wind vectors.

## 3. Results

### 3.1. Migration Peak of L. sticticalis Moths in Northern China in 2022

According to the monitoring data recorded by searchlight traps and black-light traps, the peak period of light-trap catches was recorded in late June (Figure 1a). Between 20 and 27 June, large numbers of moths were detected by light traps in Bayannur City, Ordos City, and Ulanqab City in Inner Mongolia, Zhangjiakou City in Hebei, Xinzhou City in Shanxi, and YQ in Beijing. The ovarian developmental stages of moths varied significantly across these stations (Table 1). Specifically, a total of 44,216 and 32,895 moths were detected by searchlight traps in DLTQ, Inner Mongolia, and KB, Hebei, respectively, from 23 to 26 June. On the peak day, 24,150 moths were caught in DLTQ and 21,855 in KB. From 24 to 25 June, the total catches of moths at CYQQ reached 60,320, peaking at 35,360 moths on 25 June. Additionally, 100% of the dissected females there exhibited ovarian development at III–IV stages, suggesting the presence of possible immigrant individuals. At YQ, 73% of females were at stages III–IV, thereby classifying them as immigrants. For the remaining stations, where the proportion of females with ovarian stages at III–IV was below 60%, the populations were classified as emigrants.

In black-light trap monitoring, the catches on the peak day in late June were 7382 in WQ and 1718 moths in KB of Hebei. At LH, Inner Mongolia, after moths were detected by the searchlight trap on 24 June, the black-light trap caught 4656 moths on 25 June, with 67% of females exhibiting ovarian stages III–IV, suggesting that they were partial immigrant individuals.

Overall, the main occurrence areas of *L. sticticalis* moths were centered on the border region between Inner Mongolia, northern Hebei, and northern Shanxi, with the massive landing in Inner Mongolia, followed by Hebei.

### 3.2. Simulated Trajectories of L. sticticalis in Northern China

**Figure 2:**
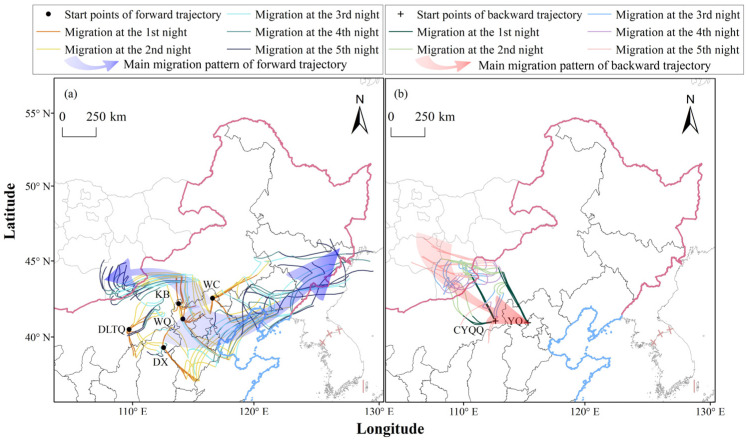
Typical forward (**a**) and backward (**b**) trajectories at representative stations of overwintering *L. sticticalis* during the peak period of light-trap catches in northern China, 2022.

It is indicated that, after emigration from 11 monitoring stations—including DX (Shanxi), WC, KB, and WQ (Hebei), XH, FZ, DLTQ, LC, WLTQQ, and WLTZQ (Inner Mongolia), and JP (Liaoning)—the potential landing areas of *L. sticticalis* were primarily concentrated in Inner Mongolia, Hebei, and Shanxi, followed by the south part of Northeast China and Mongolia (Figure 2a, Table S2). Moths from DX, WLTZQ, and WLTQQ migrated northeastward. For example, moths that took off from DX during the peak period of light-trap catches showed a 63.33% probability of moving through Hebei by the second night. By the fourth and fifth nights, the cumulative probabilities of reaching Liaoning and Jilin were 17.49% and 13.81%, respectively. Moths from LC, KB, and JP migrated northwestward. Taking KB as an example, *L. sticticalis* moths emigrating from this area had a 16.67% to 38.16% probability of entering Mongolia during their 3 to 5 nights of flight. Moths initiating their flights from WC and WQ, as well as XH and FZ, not only had the potential to cross the border into Mongolia, but also migrated northeastward. For example, those from WC could reach as far north as Heilongjiang and disperse eastward into Russia. Migrant moths from DLTQ mainly land within the autonomous region and its surrounding areas.

Based on our backward trajectory analysis, the Mongolian individuals were identified as key source populations for CYQQ and LH, and YQ (Figure 2b, Table S3). Among these, CYQQ was the primary landing zone for moths from Mongolia, with an overall probability from Mongolia of 18.26%. This was followed by YQ, which had a 2.69–6.02% probability of encountering pests from Mongolia over the second to fifth nights.

### 3.3. Simulated Trajectories and Main Landing Areas of L. sticticalis from Mongolia

Simulations of the migratory trajectories of *L. sticticalis* in northern China indicate frequent population exchanges between China and Mongolia. To further validate this conclusion, we analyzed the migratory pathways and main landing areas of Mongolian *L. sticticalis* populations based on field survey data from 50 places across 11 provinces in Mongolia: Selenge (1 station), Khentii (4), Dornod (3), Uvurkhangai (3), Bayankhongor (2), Govi-Altai (3), Bulgan (1), Tuv (23), Umnugovi (4), Dornogovi (5), and Dundgovi (1) (Figure 1b). These stations cover the perennial emergence areas of *L. sticticalis*. We also analyzed the migratory pathways and main landing areas of this pest originating from Mongolia. Monitoring data showed that *L. sticticalis* was mainly observed from early June to early August (Table 2). Adults were observed at 43 of the surveyed stations (86%), and the average moth count per hundred steps at these positive stations was 18. The highest density was recorded in Bayannuur, Bulgan Province (47.86° N; 104.90° E), with 70 moths per hundred steps on 14 June, followed by Altanbulag Soum, Tuv Province (47.88° N; 105.85° E), with 52 moths per hundred steps on the same date.

**Figure 3:**
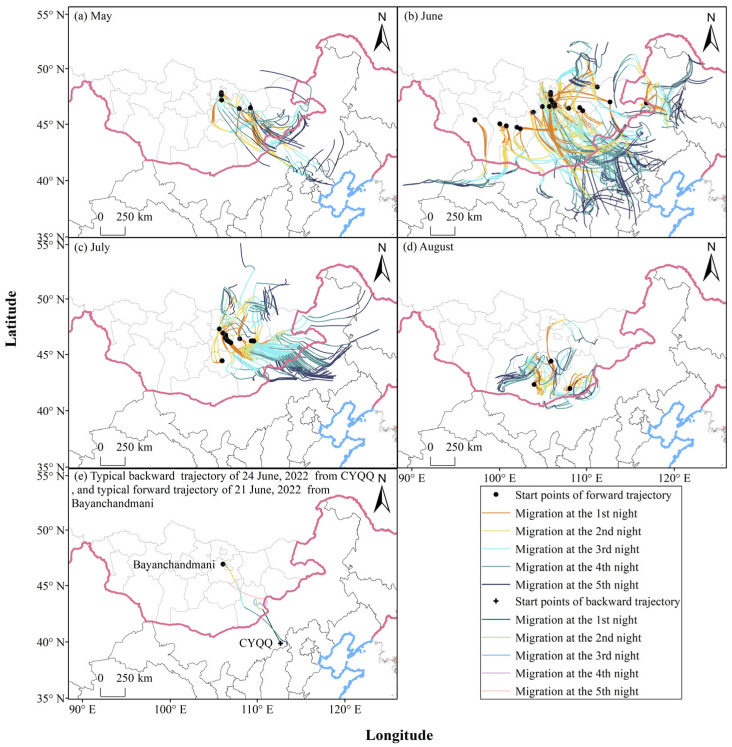
Typical forward trajectories of migratory *L. sticticalis* from June to August in Mongolia in 2022.

Further forward trajectory analysis revealed that during the occurrence period of *L. sticticalis* from June to August, the potential migratory populations monitored in Mongolia had an average 80.27% chance of entering China, compared to only 19.73% that could migrate to Russia (Table S4). The detailed probability of the *L. sticticalis* populations migrating into China ranged from 68.52% to 100%. The Inner Mongolia region of China, recording a landing probability of 73.98%, was identified as the primary landing area of Mongolia moths. Meanwhile, Hebei, Shanxi, Beijing, Jilin and Heilongjiang were the affected regions. In May and July, moths advanced into Northeast China passing through Inner Mongolia, exhibiting a distinct “cyclonic” migration pattern (Figure 3a,c). In June, the migratory paths from Mongolia were predominantly southeasterly, followed by easterly and southwesterly directions (Figure 3b). The southeasterly routes passed through Inner Mongolia, Shanxi, and Hebei, with the farthest extending to Shandong during the MTW. The easterly routes of *L. sticticalis* in June were similar to the overall trajectories in July, extending as far east as Heilongjiang. The landing probability in Heilongjiang for the actual monitoring date was 0.68%, while the probability for the total duration of the MTW was 3.91%. The southwesterly routes of *L. sticticalis* populations during the MTW could reach as far as Xinjiang (3.29%). In August, the insects originating from Mongolia only exchanged with those from Inner Mongolia, China (Figure 3d). The emigration trajectories from Mongolia clearly corresponded to the immigration trajectories in northern China. For instance, on 21 June, Bayanchandmani recorded seven moths per hundred steps. Moths emigrating from this station were computed to land in CYQQ, Inner Mongolia, on 24 June via a 4-day successive migration (Figure 3e). On the same day, the destination CYQQ experienced a massive landing of *L. sticticalis,* where 24,960 moth individuals were trapped (Table 1). Additionally, backward trajectories simulated from CYQQ on 24 June were traced back to the Bayanchandmani region.

### 3.4. Synoptic Conditions During the Peak Period of Light-Trap Catches of L. sticticalis Adults in China

#### 3.4.1. Synoptic Situation During the Peak Period of Light-Trap Catches of *L. sticticalis*

**Figure 4:**
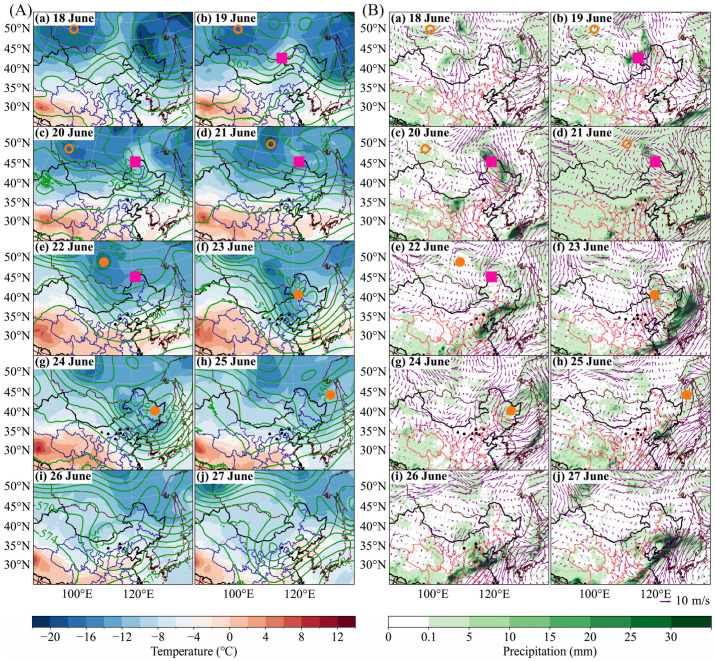
Mean nighttime temperature and geopotential height at 500 hPa (**A**), and 850 hPa wind field and accumulated precipitation (**B**) during the peak period of light-trap catches of *L. sticticalis* moths in northern China during 18 to 27 June 2022. Note: Black dots on the map indicate the start points of forward trajectories, and black triangles indicate the start points of backward trajectories. Cyclone centers are annotated on the figures, using solid circles to represent mature NCCV, solid squares for the Mongolian Cyclone, and hollow circles for the Cold center.

During 18–20 June 2022, a cold center formed to the north of the border between Xinjiang and Mongolia (Figure 4A, panels a–c). A Mongolian Cyclone formed on 19 June to the west of Northeast China, along the China–Mongolia–Russia border. From 20 to 21 June, the center of the cold air mass moved southward, but 850 hPa conditions remained affected by the Mongolian Cyclone. On 20 June, wind shear was present at WC, while on 21 June, the area was governed by southerly winds, with wind speeds around 5 m/s (Figure 4B, panels c–d). This synoptic situation significantly altered the forward trajectories from WC (Figure 2a), making the trajectory on 21 June longer than that on 20 June, with a southwest to northeast direction. On 22 June, an NCCV had formed, with its center located between 40° N and 50° N (Figure 4A, panels e). From 22 to 24 June, the NCCV intensified continuously, moving southward and squeezing the Mongolian Cyclone, eventually absorbing it on 23 June (Figure 4A, panels e–g). The rainfall zone of marked nocturnal cumulative precipitation expanded on 22 June, with the main precipitation area covering most of Shandong, eastern Inner Mongolia, central Jilin, southeastern Hebei, and western Liaoning. In these areas, nocturnal cumulative precipitation exceeded 30 mm (Figure 4B, panels e–f). By 25 June, the vortex center weakened to 550 hPa, and no considerable widespread precipitation was observed in China (Figure 4B, panels h). Influenced by the development of the NCCV, from 22 to 23 June, the main occurrence areas of *L. sticticalis* moths were dominated by northwesterly winds at the 850 hPa level, while from 24 to 25 June, the region experienced southwesterly winds (Figure 4B). Taking the forward trajectory from DX (Figure 2a) and the backward trajectory from CYQQ (Figure 2b) as examples, regardless of the departure date, the trajectories from 22 to 23 June all show a northwest-to-southeast direction, while those from 24 to 25 June all follow a southwest-to-northeast direction. From 25 to 27 June, the influence of the NCCV gradually weakened. North of the border between Xinjiang and Mongolia, a shortwave trough formed (Figure 4A, panels h–j). This caused considerable shifts in trajectory directions around 25 June for stations like WQ (Figure 2a). After takeoff, moths initially migrated eastward under the influence of the NCCV and northwesterly winds on 24–25 June. On 26–27 June, they then shifted northward or even northwestward due to the southwesterly winds brought by the shortwave troughs. After the shortwave trough caught up with the cold vortex on 26 June, precipitation increased by at least 5 mm in North China and central Inner Mongolia (Figure 4B, panel i), which could explain the capture of 18,816 moths in WQ (Table 1).

In summary, the migratory trajectories of *L. sticticalis* populations at most monitoring stations closely corresponded to the development of the Mongolian Cyclone and the NCCV. Moreover, the peak period of light-trap catches in northern China during this study was primarily driven by the NCCV.

#### 3.4.2. Specific Meteorological Mechanisms Affecting the Migration and Landing of *L. sticticalis* Moths on Peak Days

By analyzing the synoptic conditions on peak days and extracting relevant meteorological elements (including wind speed, wind direction, temperature, downdrafts, and precipitation), the meteorological mechanisms contributing to the concentrated landing of *L. sticticalis* in northern China in late June 2022 were further clarified. As shown in Table 3, the average nocturnal wind speed at 850 hPa during the peak days in northern China was 3.3 ± 2.3 m/s, with a mean wind direction of 171.4°, indicating prevailing weak southerly winds across the monitoring stations. However, on 23 June, wind speeds increased significantly in WLTZQ and DLTQ (Inner Mongolia). JP (Liaoning) had mean nighttime wind speeds of 9.7 m/s. This period coincided with the vigorous phase of the NCCV, which made it easier for *L. sticticalis* to migrate vast distances north and wreak havoc. Additionally, the average nocturnal temperature at 850 hPa across the monitoring stations in late June was 22.6 ± 4.4 °C, above the low temperature threshold for *L. sticticalis* flight. This indicates that temperature did not restrict migratory activity during this period. On nights without precipitation, downdrafts were observed at 71.43% of the stations. The maximum vertical velocity occurred in DLTQ (Inner Mongolia) on 23 June, reaching 23.7 × 10^−2^ Pa/s. Regarding precipitation, WC (Hebei) experienced moderate rain on the night of 21 June. Between 26 and 27 June, four sites—LC (Hebei), WQ (Hebei), YQ (Beijing), and DLTQ (Inner Mongolia)—experienced rainfall events, mainly moderate rain. In summary, downdrafts and precipitation were identified as the primary meteorological causes for the concentrated landing of *L. sticticalis* in northern China.

### 3.5. Interactive Effects of the NCCV and Mongolian Cyclone During the Occurrence Period of L. sticticalis Moths

In northern China and Mongolia, the NCCV and the Mongolian Cyclone interact and influence each other. A cold trough prior to the formation of the cold vortex often triggers Mongolian Cyclones. These processes collectively disrupt the meteorological background (Figure S5), thereby influencing the migration and landing of *L. sticticalis*. This study systematically examined the occurrence processes of the NCCV and the Mongolian Cyclone, along with their interactive effects, to further investigate the atmospheric circulation patterns governing the extensive migration and collective landing of *L. sticticalis* from 28 May to 13 August 2022. During this period, there were seven NCCV events and three Mongolian Cyclone events. Among the NCCV processes, three were classified as Central Vortex (40–50° N) and four as Northern Vortex (50–60° N), with no Southern Vortex (35–40° N) processes observed (Table 4).

**Figure 5:**
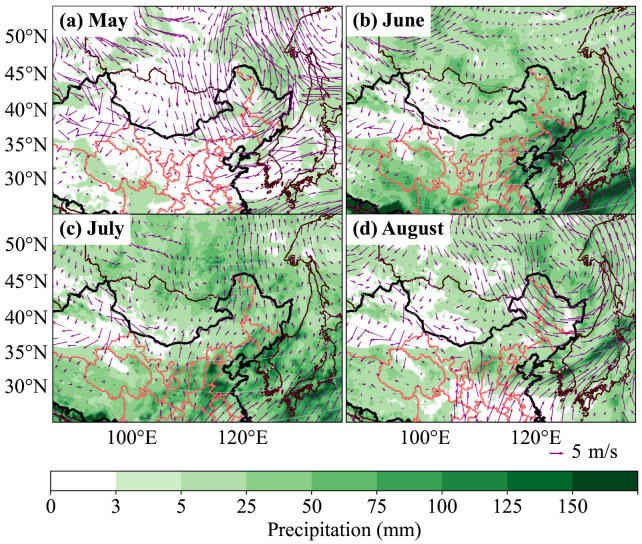
Average nighttime wind field at 850 hPa and accumulated precipitation of *L. sticticalis* from May to August in 2022.

From 28 to 31 May, the migration trajectories from central and eastern Mongolia, influenced by Cold Vortex No.12 (Figure 5a), exhibited a “cyclonic” pattern (Figure 3a). Cyclonic activity was most pronounced in June, with two Mongolian Cyclones and three NCCV processes recorded, including three Central Vortex processes and one Northern Vortex process. The frequent activity and alternation of these two systems, covering the entire month of June, resulted in a situation where both cyclones coexisted and were relatively strong (Figure 4). Throughout June, the 850 hPa level along the China–Mongolia border was consistently dominated by northerly–northwesterly winds at night, while southerly winds in North China were less obstructive than those in other months (Figure 5b), which provided favorable transport conditions for the migration of Mongolian *L. sticticalis* into China. Precipitation in the border zone totaled less than 25 mm for the month, while that in northern Shanxi, Hebei, and Beijing exceeded 25 mm, and southern Hebei and Shandong saw over 75 mm of precipitation (Figure 5b). Consequently, part of the populations in central and eastern Mongolia migrated southeastward through Inner Mongolia, reaching as far as Shandong, while another part was carried into Northeast China by the “cyclonic” wind field (Figure 3b). Populations taking off from western Mongolia were simulated to enter northern Gansu and then move westward to Xinjiang under the influence of weak wind shear, with less than 5 mm of precipitation during this period (Figure 5b). In early to mid-July, the Central Vortex, Northern Vortex, and Mongolian Cyclones occurred in succession, influencing the weather conditions. During this period, populations from central and eastern Mongolia were carried into Northeast China by the “cyclonic” wind field (Figure 3c). No cold vortices or Mongolian Cyclones developed in late July. Marked wind shear and strengthened wind fields were observed along the border in July, with prevailing southerlies over northern China and northerlies over Mongolia. This pattern not only enhanced cross-border insect source exchange but also facilitated the concentrated landing of airborne *L. sticticalis* (Figure 5c). Concurrently, monthly cumulative precipitation exceeded 25 mm in Shanxi, Hebei, Northeast China, and eastern Inner Mongolia (Figure 5c), providing favorable conditions for moth landing. In the first half of August, one Northern Vortex process was recorded, but no Mongolian Cyclones formed, leading to short but concentrated migration pathways with diverse directions. (Figure 3d). During this period, sustained northerly–northwesterly winds prevailed over Mongolia along the border, while adjacent areas in China experienced southerly winds (Figure 5d), together leading to massive landings of Mongolian *L. sticticalis* along the border region.

As shown in Figure 5, during the estimated emigration period of Mongolia *L. sticticalis* adults from May to August 2022, the northerly winds prevailed over Mongolia, and southerly winds were predominant in northern China. The persistent wind shear along the China–Mongolia border, in terms of its spatiotemporal distribution and intensity, played a decisive role in shaping the migratory pathways, distance, and landing zones of *L. sticticalis*. The NCCV and Mongolian Cyclone systems, which gave rise to and controlled this atmospheric circulation pattern, along with their interactions, were identified as the main reasons for this pest migrating from Mongolia to northern China during the study period.

## 4. Discussion

### 4.1. Notable Exchange of Insect Populations Between China and Mongolia

Since the beginning of the 21st century, it has been widely recognized that, in addition to local overwintering populations, insects colonizing Northeast China are partially derived from Mongolia and Russia [6,16,17,19]. Frolov [20] classified the *L. sticticalis* populations in China and Mongolia as one Central Asian system. Data from a field survey conducted in 2022 identified that primary infestation areas were mainly located in the border areas of Inner Mongolia, northern Hebei, and northern Shanxi in China, as well as in central and eastern Mongolia (Figure 1). This spatial distribution is closely related to the region’s ecological conditions. *L. sticticalis* is mostly found in China north, of the 12 °C mean annual temperature isotherm, and in areas with yearly precipitation of 100–700 mm. The worst damage happens in the 2nd–3rd generation occurrence zones, which have a mean annual temperature between 0 and 8 °C, making them favorable for the pest to overwinter [63,64]. This area lies in the temperate dry and semi-arid zone of China [65], with most of the land covered in grasslands and desert steppes, and a higher coverage of forestland cropland in the eastern region [65,66]. Likewise, the infestations of *L. sticticalis* in Mongolia are found in temperate dry or semi-arid temperature zones. Most of Mongolia’s terrain is covered in grasslands and desert steppes, but there are also some forest steppes in the north [67,68], which are generally less disturbed by humans. In 2022, the average January temperatures of all monitoring stations in Mongolia were above the average supercooling point for diapausing *L. sticticalis* larvae (−26.8 °C) [69]. The mean annual temperature range was between −1.2 °C and 7.6 °C, allowing development for 1–3 generations of the pest per year [63]. The average annual precipitation across the stations was 278.7 mm, with 86.05% of the stations meeting the annual precipitation criteria (100–700 mm) for suitable *L. sticticalis* habitats. During the 2022 MTW, 54.55% of the stations met the precipitation requirement for medium-to-high suitability (15–50 mm) (Table S5) [18]. In conclusion, the core outbreak areas in both China and Mongolia share high consistency in climate types, underlying surface composition, and key meteorological factors, together forming core infestation areas for *L. sticticalis* in both countries.

Based on survey data from the two core areas, this study conducted trajectory simulations. The forward trajectory results showed that populations from North China (e.g., DX in Shanxi) primarily migrated northeastward towards Northeast China, while populations from North China (exemplified by KB in Hebei) and Inner Mongolia also migrated north–northwestward, towards the China–Mongolia border. Backward trajectory analysis indicated that the principal sources of *L. sticticalis* populations in northern China included Hebei, Inner Mongolia, and Mongolia. At the same time, the potential emigration trajectories of Mongolia showed that *L. sticticalis* from Mongolia may go directly southeast into China after departure if the weather is good. In addition, the NCCV and the Mongolian Cyclone could induce the population observed in Mongolia to take another two migratory routes: one towards the east and one towards the southwest. These routes extended as far south as northwestern Shandong, north to northern Heilongjiang, and west to southern Xinjiang. Notably, in late June 2022, the emigration paths from Mongolia clearly aligned with the backward trajectories of populations in northern China (Figure 3e). Our findings are consistent with the inferences drawn from the backward trajectories in North China by Chen et al. [6]. Chen et al. [6,16] pointed out that an immigration of *L. sticticalis* occurred in Northeast China in late May and early June 2001, which was earlier than the occurrence of populations in North China, suggesting that these adults are highly likely to have come from northeastern Mongolia. Furthermore, in 2002 and 2007, the insects in Northeast China mainly originated from eastern Mongolia, and their migration paths closely aligned with the identified emigration paths from Mongolia in late May and early June [6]. The migratory path along the border areas of Shanxi, Inner Mongolia, and Hebei in early August 2003 also coincided with the emigration path from southern Mongolia during the period described here [16]. Additionally, historical occurrence dynamics of migratory *L. sticticalis* (Table S1) and relevant literature [45,70,71] have also been referenced to support our research. Previous studies [70,71] indicate that the peak migration period for *L. sticticalis* populations in northern China occurs from June to August. It is also suggested that there is frequent immigration of foreign populations and emigration of local populations in northern China in late June (Table S1). This aligns with the migratory population dynamics of *L. sticticalis* monitored in 2022. Compared with the field survey data collected in Mongolia, the dataset from northern China was limited to late June. This temporal constraint may have led to an underestimation of the probability of population exchange between China and Mongolia, particularly the likelihood of emigration from China to Mongolia—because peak migration events in northern China outside late June would not have been captured.

Ovarian development serves as an important physiological indicator for determining characteristics of migratory insects and has been applied in studies on relevant species [55,56,57,58]. According to long-term population monitoring data, the sustained presence of only individuals with low ovarian development stages suggests the emigration of the local population. In contrast, a prominent appearance of high-grade individuals amidst declining local population density typically indicates immigration from external sources. When the proportion of low-grade individuals gradually decreases while the number of high-grade individuals consistently increases, this generally indicates the natural development of a local population [59]. However, restricted by limited data in 2022, this study could not undertake long-term monitoring or systematic ovarian dissection at all of the monitoring stations, and thereby failed to thoroughly characterize the population dynamics of *L. sticticalis* populations. In future work, it is proposed to use systematic monitoring data and the daily composition of ovarian developmental stages as a biologically grounded diagnostic tool to distinguish emigration, immigration, and local population dynamics, which could provide critical biological insights that enhance the understanding of migration patterns of *L. sticticalis*.

Cheng et al. [41] reported that female moths of *L. sticticalis* predominate in catches from both searchlight and black-light traps. Thus, a criterion based on ovarian development stage is a useful indicator in this study. Specifically, a population in which ≥65% of female moths are at ovarian stages III–IV is consistent with an immigrant cohort. Moreover, in this study, for the purposes of our migration trajectory modeling, we treated individual *L. sticticalis* as a passive particle, thereby simplifying the representation of movement and not explicitly incorporating absolute population size or density into the model.

Radar study revealed that the migration direction of *L. sticticalis* aligns with the airflow direction [24], which prompted the application of the HYSPLIT model on migration routes of this insect pest. However, HYSPLIT modeling has certain limitations, as the outputs do not directly illustrate the effects of topography on the migration routes of *L. sticticalis* moths, and it lacks considerations of self-powered flight behavior and its quantitative response to specific meteorological factors or conditions. There is a certain degree of difference between the simulation results and the actual insect migration. Thus, multi-source data, including long-term population dynamics, migratory behaviors, and environmental responses, collected from mark-release-recapture experiments [15] and radar monitoring networks [44], will improve the accuracy of simulated migration pathways and enhance predictive modeling for regional insect outbreak management. In future studies, we should also prioritize methods such as pollen metabarcoding [72] and DNA sequencing [73], which can provide valuable insights into the biological tracking of moth migration. The significant role of topography in shaping migration patterns of insects has been well established [53,74,75]. Additionally, the impact of moonlight [76] and the free flight activity of *L. sticticalis* [77] on light trapping methods should be considered in the future.

### 4.2. Noteworthy Impact of the NCCV

Meteorological analysis further revealed that the overall near-surface wind fields in June and July were highly conducive to the invasion of *L. sticticalis* from Mongolian source regions and the insect source exchange between Mongolia and Russia. Notably, during June, when the NCCV and the Mongolian Cyclone frequently interacted, an additional southwesterly migration route was generated, originating from western Mongolia, extending as far as southern Xinjiang in China. Existing surveys indicate that Xinjiang is a high-altitude occurrence area for *L. sticticalis* in China, with insects there primarily affecting Hotan in southern Xinjiang, as well as areas bordering Kazakhstan, including Altay, Bole, and Tacheng in northern Xinjiang [78,79]. In southern Xinjiang, the peak abundance period of overwintering-generation adults typically occurs in early July [80]. Therefore, substantial numbers of *L. sticticalis* migrating from Mongolia into Xinjiang via this southwesterly route (“Mongolia–Xinjiang, China”) in June supplement the local population base and potentially exacerbate outbreak threats in some areas. Moreover, the average nighttime wind patterns during the occurrence period of *L. sticticalis* moths in late May and early to mid-August 2022 showed that cyclonic systems changed the atmospheric conditions responsible for *L. sticticalis* migration, making it easier for insects to move between central and eastern Mongolia and China. Previous studies have confirmed that both the Mongolian Cyclone [39] and the NCCV [15,81] play major roles in the migration process of insects. The near-surface wind direction associated with these systems influences migration direction, while the wind speed determines migration distance. Precipitation and downdrafts triggered by these cyclonic systems can also lead to massive landings of *L. sticticalis* [16,82]. In this study, we found that from June to August 2022, the NCCV relatively dominated the synoptic patterns. This is because the NCCV develops deeply over Northeast China, Inner Mongolia, and Mongolia, exerting substantial and prolonged effects on local wind fields [22,34,35]. The changes in weather conditions and meteorological elements caused by the NCCV’s interaction with and influence over the Mongolian Cyclone substantially affected the migration pathways, distances, and the occurrence of landings of *L. sticticalis* in that year. Moreover, by leveraging these specific weather conditions, *L. sticticalis* from Russia could also migrate into China and cause damage. Given suitable wind fields and the presence of substantial source populations in Russia and Mongolia, China faces an ongoing risk of successive immigration and potential outbreaks of *L. sticticalis* [12,19,83]. In conclusion, our findings underscore the regulatory role of mid- to high-latitude cyclonic systems in East Asia on the cross-border migration of *L. sticticalis* between China and Mongolia.

### 4.3. Effects of Climate Change and Extreme Rainfall Events on the Migration of L. sticticalis

Temperature and precipitation have a significant effect on the migration of *L. sticticalis*. The optimal temperature for migration is 22 degrees Celsius [27]. Adults will not fly if the temperature is below 15 °C, but they tend to fly significantly more if the temperature is over 20 °C [8,28]. Currently, the main occurrence areas of *L. sticticalis* in China—North China, Northeast China, and Northwest China—are experiencing increasing surface temperatures [84], which may lead to an earlier emergence of *L. sticticalis* [64]. Monitoring data from the Pest Forecasting Division of the National Agro-Tech Extension and Service Center confirmed that, in 2022, the first appearance of overwintering adults monitored by light traps occurred earlier than that in average years in most parts of China [85]. By late April to mid-May, moths had been detected at the monitoring stations across various provinces in northern China [85]. However, the peak period of light-trap catches of the *L. sticticalis* overwintering generation was in mid- to late June 2022, which was slightly later than that in average years [86]. This potential delay might be attributed to the influence of the migrant population from Mongolia. In Mongolia, the number of moths per hundred steps in the field showed two emergence peaks: early to mid-June and early to mid-July (Table 2). In 2022, the infestation was less severe in China than in a typical outbreak year [12]. One probable factor for this is comparatively higher precipitation in the predominant infestation areas in that year. For instance, the end of the second and third outbreak cycles of *L. sticticalis* in China coincided with periods of low temperatures, heavy rainfall, and severe adverse weather conditions [81]. In recent years, extreme precipitation events have become more frequent [84], showing a significant increase in precipitation intensity in the Northeast and North China (about 6–12%) [87]. Narrowing temperature and precipitation disparities between northern and southern China [87] may facilitate the northward migration of *L. sticticalis*. Cyclonic systems are also undergoing changes. For example, the frequency of extratropical cyclones (such as the Mongolian Cyclone) has decreased during summer, but their intensity has increased [88]. Moreover, the NCCV has shown a tendency to shift northward in May [89]. These changes are likely to influence the migration patterns of *L. sticticalis*.

### 4.4. Relationship Between Agricultural Landscape Changes and L. sticticalis Outbreaks

Some researchers, based on the cyclical patterns of *L. sticticalis* outbreaks and field population monitoring data, along with solar activity cycles, have inferred that the fourth outbreak cycle of *L. sticticalis* occurred approximately between 2016 and 2025 [11,90]. Although the population levels remained low in 2016 and 2017 [9,91], a resurgence was observed between 2018 and 2019 [10,11], culminating in a typical severe outbreak year in 2020 [12], marking the entry into the fourth outbreak cycle. *L. sticticalis* has mostly stayed at moderate levels in recent years, without major outbreaks, thanks to timely monitoring, early warnings, and good control. Areas including Inner Mongolia, Hebei, and Northeast China, where major food crops are cultivated, are at potential risk of severe infestations [92]. Hebei and Shanxi, located in the climatically suitable Yellow River basin, feature vast areas of staple crops like corn and millet. In the majority of the 96 banners and counties in Inner Mongolia, primary host crops of *L. sticticalis*, including wheat, corn, potatoes, and soybeans, are grown. The principal cultivation regions of soybeans and potatoes correspond with the primary occurrence zones of *L. sticticalis* identified in this study [93]. Mongolia shares similar land cover types with northern China, with its agricultural structure primarily focused on livestock farming, although wheat (a staple food crop), potatoes, and other crops are also grown [18]. Adjustments in the planting structure of host plants have influenced the migration and occurrence patterns of *L. sticticalis* during its fourth cycle [11,90]. *L. sticticalis* is polyphagous but exhibits distinct feeding and oviposition preferences. Its preference for soybeans and potatoes is significantly higher than for corn. During oviposition, adults lay significantly more eggs on leguminous forages than on gramineous forages [94]. Survey data from the China Agricultural and Rural Information Network (http://www.agri.cn/ accessed on 6 April 2025) indicate that Heilongjiang and Inner Mongolia were major soybean-producing provinces in 2022. Heilongjiang, Jilin, Inner Mongolia, Henan, Liaoning, Shanxi, Gansu, and the Ningxia Hui Autonomous Region are key corn-growing areas. Soybean production has increased steadily year by year since 2015, while corn production remained roughly the same. The distribution of crops significantly influences infestation degrees and areas. Since agricultural structure adjustments, the environment has become more conducive to the migration, survival, and reproduction of *L. sticticalis*. Therefore, sustained and close monitoring of *L. sticticalis* population dynamics in China holds considerable importance for guiding early warning and sustainable management of this major pest in the context of evolving agricultural landscapes.

## 5. Conclusions

In 2022, the principal infestation areas of *L. sticticalis* were located in the border regions of Inner Mongolia, northern Hebei, and northern Shanxi in China, in addition to central and eastern Mongolia, with frequent exchanges between populations in China and Mongolia. The migrating populations from Mongolia followed three main pathways: a predominant southeasterly route, with supplementary eastward “cyclonic” and southwesterly paths. The main landing areas in China were North China and Northeast China, with migration range extending as far as Shandong, Heilongjiang, and Xinjiang. Populations from North China (such as DX in Shanxi) primarily migrated northeastward toward Northeast China, while populations from North China (exemplified by KB in Hebei) and Inner Mongolia migrated north–northwestward, heading toward the China–Mongolia border. Insect sources from North China could reach Northeast China and Mongolia after 1–5 consecutive nights of migration. Meteorological systems played a major regulatory role in the cross-border migration of *L. sticticalis*. From late May to early August 2022, there were seven NCCV events and three Mongolian Cyclone events. During the migration period, the interactions between these systems determined the synoptic circulation. Their spatial and temporal distribution and intensity were very important at the very stage when the pathways were established, and the range of the cross-border migration was expanded. Persistent wind shear at the China–Mongolia border made it easier for insects to interact between the two areas. Wind fields of prevailing NCCV events provided the driving force for the immigration from Mongolia into China and for the northeastward migration of populations from North China, contributing to the risk of infestation. Moreover, the precipitation and downdrafts triggered by the NCCV were key factors driving the concentrated landing of *L. sticticalis* populations in northern China.

## Figures and Tables

**Table 1:** Light-trap monitoring of overwintering *L. sticticalis* in northern China, 2022.

Province	Monitoring Station	Longitude/°	Latitude/°	Peak Period of Light-Trap Catches	Peak Day	Peak Light-Trap Catches	Total Catches of Trapped Moths	Proportion of Female Moths with Ovaries at Stage III–IV on Peak Day	Light-Trap Type
Inner Mongolia	WLTZQ	107.16	41.07	22 to 25 June	23 June	9286	22,672	10%	Searchlight trap
	LH	107.4	40.75	24 to 26 June	/	/	9328	/	Searchlight trap
	WLTQQ	108.65	40.72	21 to 26 June	/	/	6345	52%	Searchlight trap
	DLTQ	110.03	40.4	23 to 26 June	23 June	24,150	44,216	22%	Searchlight trap
	CYQQ	113.221	40.7788	24 to 25 June	25 June	35,360	60,320	100%	Searchlight trap
	XH	113.88	40.88	20 to 25 June	25 June	5640	21,951	40%	Searchlight trap
	FZ	113.11	40.437	22 to 26 June	25 June	4700	20,600	30%	Searchlight trap
	LC	112.496	40.534	25 to 26 June	26 June	5812	11,012	50%	Searchlight trap
Shanxi	DX	112.95	39.07	21 to 25 June	24 June	6700	16,000	/	Searchlight trap
Hebei	WC	117.72	41.95	20 to 25 June	21 June	701	1420	45%	Searchlight trap
	KB	114.62	41.85	23 to 26 June	25 June	21,855	32,895	40%	Searchlight trap
	WQ	114.87	40.82	21 to 27 June	26 June	18,816	31,688	10%	Searchlight trap
Beijing	YQ	116.07	40.46	24 to 27 June	27 June	5195	6648	73%	Searchlight trap
Liaoning	JP	119.65	41.4	25 to 27 June	25 June	869	1158	/	Searchlight trap
Inner Mongolia	LH	107.4	40.75	21 to 25 June	25 June	4656	7818	67%	Black-light trap
	WLTQQ	108.65	40.72	24 to 26 June	/	/	6191	53%	Black-light trap
	DLTQ	110.03	40.4	24 to 26 June	26 June	589	1410	35%	Black-light trap
	XH	113.88	40.88	/	25 June	657	/	35%	Black-light trap
	LC	112.496	40.5335	24 to 26 June	25 June	545	1302	40%	Black-light trap
Shanxi	DX	112.95	39.07	22 to 25 June	25 June	243	327	/	Black-light trap
Hebei	KB	114.62	41.85	23 to 26 June	25 June	1718	2470	40%	Black-light trap
	WQ	114.87	40.82	23 to 27 June	26 June	7382	10,000	10%	Black-light trap

Note: the symbol/indicates ‘Not recorded’.

**Table 2:** Field survey data of *L. sticticalis* in Mongolia from June to August, 2022.

Province	Monitoring Station	Longitude/°	Latitude/°	Elevation/m	Number of Moths per Hundred Steps	Survey Date	Estimated MTW
Selenge	Baruunharaa soum	106.04181	48.9493	805	45	3 June	30 May to 7 June
Khentii	Moron soum	110.34028	47.38178	1113	7	1 July	27 June to 5 July
Khentii	on the way to Chingis city (Poa sp.)1	110.03486	47.42117	1029	7	13 July	9 to 17 July
Khentii	on the way to Chingis city (Raps)2	109.97364	47.42033	1164	2	13 July	9 to 17 July
Khentii	6 km to the south from Tsenkhermandal soum	109.95883	47.6761	1182	23	1 June	28 May to 5 June
Dornod	Bulgan soum	114.09788	48.00692	767	7	11 June	7 to 15 June
Dornod	Khalkh gol soum, Tultiin hyasaa	118.99038	47.52393	740	15	13 June	9 to 17 June
Dornod	Bayanuul soum, Uikhan	112.54358	49.47246	859	18	5 June	1 to 9 June
Uvurkhangai	Elsen tasarkhai	103.673624	47.34378		29	10 June	6 to 14 June
Uvurkhangai	Khairkhandulaan soum	101.97989	45.80635	1749	10	10 June	6 to 14 June
Uvurkhangai	Nariin teel soum	101.52296	45.94305	1961	22	10 June	6 to 14 June
Bayankhongor	Station1	100.11878	46.03059	1792	4	11 June	7 to 15 June
Bayankhongor	Bombogor soum, Baidragiin guur	99.26623	46.17355	1557	15	11 June	7 to 15 June
Govi-Altai	Station2	95.93209	46.36514	1740	2	13 June	9 to 17 June
Govi-Altai	Sharga soum	95.29187	46.23174	1002	12	15 June	11 to 19 June
Govi-Altai	Tonkhil soum	94.04129	46.18017	2029	6	20 June	16 to 24 June
Bulgan	on the way to Bayannuur	104.901206	47.85859		70	14 June	10 to 18 June
Tuv	Baganuur, Kherlen river	108.44704	47.66481	1240	15	2 June	29 May to 6 June
Tuv	Bayanchandmani sum	106.29135	48.22033		7	21 June	17 to 25 June
Tuv	River Kherlen	108.44704	47.66481	1290	12	30 June	26 June to 4 July
Tuv	Altanbulag soum, on the way to Khustai	105.845756	47.878617		52	14 June	10 to 18 June
Tuv	Khustain davaa	106.04611	49.15895	934	28	4 June	31 May to 8 June
Tuv	Dugan khad	106.06647	48.48056	1241	34	4 June	31 May to 8 June
Tuv	Nearby Ulaanbaatar1	106.56962	48.06569	1291	15	4 July	30 June to 8 July
Tuv	Nearby Ulaanbaatar2	106.54774	47.89773	1511	12	4 July	30 June to 8 July
Tuv	Nearby Ulaanbaatar3	106.53195	47.88539	1389	6	4 July	30 June to 8 July
Tuv	Nearby Ulaanbaatar4	106.53997	47.87461	1392	38	4 July	30 June to 8 July
Tuv	Nearby Ulaanbaatar5	106.56419	47.81877	1261	21	5 July	1 to 9 July
Tuv	Nearby Ulaanbaatar6	106.50265	47.79125	1245	5	5 July	1 to 9 July
Tuv	Nearby Ulaanbaatar7	106.53363	47.72104	1284	17	5 July	1 to 9 July
Tuv	Nearby Ulaanbaatar8	106.62505	47.69783	1288	42	5 July	1 to 9 July
Tuv	Nearby Ulaanbaatar9	106.68345	47.65949	1328	25	5 July	1 to 9 July
Tuv	Nearby Ulaanbaatar10	106.73286	47.58898	1369	11	5 July	1 to 9 July
Tuv	Nearby Ulaanbaatar11	106.73288	47.55789	1456	15	6 July	2 to 10 July
Tuv	Nearby Ulaanbaatar12	106.90522	47.46908	1616	21	6 July	2 to 10 July
Tuv	Nearby Ulaanbaatar13	107.04022	47.4083	1528	28	6 July	2 to 10 July
Tuv	Nearby Ulaanbaatar14	107.05425	47.40585	1520	9	6 July	2 to 10 July
Tuv	Nearby Ulaanbaatar15	107.25216	47.31834	1447	18	6 July	2 to 10 July
Tuv	Jargalant1	105.68692	48.61168	1101	12	19 July	15 to 23 July
Tuv	Jargalant2	106.18946	48.22996	1188	18	19 July	15 to 23 July
Umnugovi	Tsogtsetsii	103.85126	43.56624	2184	8	3 August	30 July to 7 August
Umnugovi	on the way to Khanbogd	106.70994	43.03794	1241	0	4 August	
Umnugovi	Khanbogd, Demchigiin khiid	107.03578	43.11028	1215	0	4 August	
Umnugovi	Khanbogd	107.40234	43.33004	977	0	5 August	
Dornogovi	on the way to Khatanbulag1	108.05357	43.29703	847	0	5 August	
Dornogovi	on the way to Khatanbulag2	108.39922	43.16482	1016	2	5 August	1 to 9 August
Dornogovi	Khovsgol, Ergeliin zoo	109.21003	43.32083	1016	0	6 August	
Dornogovi	on the way to Zuunbayan	109.2626	43.69572	829	0	7 August	
Dundgovi	Station3	106.05296	45.68972	1457	3	2 August	29 July to 6 August
Dornogovi	Station4	109.38435	44.78996	999	0	7 August	

**Table 3:** Average nighttime meteorological factors at stations on peak days of *L. sticticalis* caught by light traps in northern China.

Monitoring Station	Peak Day	850 hPa Wind Speed/(m/s)	850 hPa Wind Direction/°	850 hPa Temperature/°C	Accumulated Precipitation/mm	850 hPa Vertical Velocity/(×102 Pa/s)	Precipitation Class	Light-Trap Type
WLTZQ	23 June 2022	5.8	178 (S)	24.4	0	−16.6		Searchlight trap
DLTQ	23 June 2022	5.8	106.1 €	24.2	0	23.7		Searchlight trap
DLTQ	26 June 2022	2.3	171.2 (S)	17.9	1.1	4.4	Light rain	Black-light trap
LH	25 June 2022	4.5	155 (SE)	28.6	0	−22.7		Black-light trap
CYQQ	25 June 2022	1.3	216.8 (SW)	25.2	0	3.2		Searchlight trap
XH	25 June 2022	1.8	199.4 (S)	25.6	0	3.8		Search and black-light trap
FZ	25 June 2022	1.4	231.4 (SW)	25	0	1.2	Drizzle	Searchlight trap
LC	25 June 2022	1.2	286.8 (W)	28	0	2.1	Drizzle	Black-light trap
LC	26 June 2022	1.1	60.8 (NE)	17.3	5.9	−1.4	Moderate rain	Searchlight trap
DX	24 June 2022	2.2	267.6 (W)	26.8	0	8.1		Searchlight trap
DX	25 June 2022	1.2	294.9 (NW)	28	0	10.6	Drizzle	Black-light trap
WC	21 June 2022	2.1	240 (SW)	16.6	11.8	−1	Moderate rain	Searchlight trap
KB	25 June 2022	3.3	20.3 (N)	20.9	0	5.8		Search and black-light trap
WQ	26 June 2022	3.7	208.1 (SW)	17.4	11.7	−11.6	Moderate rain	Search and black-light trap
YQ	27 June 2022	5.2	21.7 (N)	16.4	0.5	−20.6	Light rain	Searchlight trap
JP	25 June 2022	9.7	83.6 (E)	18.6	0.7	2.2	Light rain	Searchlight trap

Note: positive vertical velocity indicates downward air motion.

**Table 4:** Occurrence of Cyclones from late May to early August 2022.

Cyclonic System	Number	Serial Number	Type	Occurrence Time
The Northeast China Cold Vortex	1	12	Northern vortex	27 May–1 June
	2	13	Central vortex	4–6 June
	3	14	Central vortex	8–14 June
	4	15	Northern vortex	22–25 June
	5	16	Northern vortex	8–10 July
	6	*	Central vortex	16–19 July
	7	*	Northern vortex	7–11 August
The Mongolia cyclone	1	/	/	1–5 June
	2	/	/	19–22 June
	3	/	/	12–16 July

The serial numbers are cited from the Liaoning Meteorological Service (http://ln.cma.gov.cn/xwzx/ztbd/dblwjcybgb/index.html accessed on 5 December 2024). The * symbol denotes the absence of officially published records. The NCCV is classified into southern (35–40° N), central (40–50° N), and northern types (50–60° N) [22]. The Mongolia cyclone is subjectively identified based on forecast indicators [62].

## Data Availability

The meteorological data supporting the results of this study are publicly available on the open-access website https://cds.climate.copernicus.eu/ [accessed on 16 December 2024], as mentioned in the Section 2.2. The insect data are presented in the Section 2.1. For further inquiries regarding the data or additional information, please contact the corresponding author.

## Supplementary Materials

The following supporting information can be downloaded at: https://www.mdpi.com/article/10.3390/insects17020218/s1, Table S1: Possible valid source areas of *L. sticticalis* in northern China documented in recent years; Table S2: Probabilities of endpoints of forward trajectory of overwintering *L. sticticalis *in China at the peak of emergence, 2022; Table S3: Probabilities of endpoints of backward trajectory of overwintering *L. sticticalis* in China at the peak of emergence, 2022; Table S4: Probabilities of endpoints of forward trajectory of *L. sticticalis* from Mongolia during May to August, 2022; Table S5: Habitat suitability in the core areas of Mongolia in 2022; Figure S1: The forward (a) and backward (b) migration trajectories of overwintering *L. sticticalis* moths during the peak period of light-trap catches in northern China, 2022; Figure S2: The forward flight trajectories of migratory *L. sticticalis* moths from June to August in Mongolia, 2022; Figure S3: Untrimmed plots of all possible results: forward (a) and backward (b) trajectory point plots of overwintering *L. sticticalis* during the peak period of light-trap catches in northern China, 2022; Figure S4: Daily average nighttime wind field at 850 hPa and accumulated precipitation of *L. sticticalis* at representative stations at typical stations during the dates of peak light-trap catches emergence peak, 2022; Figure S5: Average nighttime temperature and geopotential height at 500 hPa (a,c,e) and 850 hPa (b,d,f) during the typical period of the NCCV affecting the Mongolian Cyclone from May to August in 2022. a and b are before the cold vortex absorbed the Mongolian Cyclone on 22 June; (c,d) are the Mongolian Cyclone spawned by the trough behind the NCCV on 12 July; (e,f) are troughs before the cold vortex, giving rise to a cyclone on 4 August. Cyclone centers were annotated on the figures, with solid circles for the mature NCCV, solid squares for the Mongolian Cyclone, and hollow circles for the cold center. [95]

## Abbreviations

The following abbreviations are used in this manuscript:

*L. sticticalis*	*Loxostege sticticalis*
NCCV	Northeast China Cold Vortex
HYSPLIT	Hybrid Single-Particle Lagrangian Integrated Trajectory
AGL	Above ground level
MTW	Migration time window
