# Supplementary material for: Aerial Trajectories and Meteorological Drivers of Transboundary Loxostege sticticalis Migration Across Northern China and Mongolia, 2022
Source: Insects. 2026 Feb 19;17(2):218. doi: 10.3390/insects17020218 (PMC12941310; doi:10.3390/insects17020218)
Supplement: Supplementary file 1 [file insects-17-00218-s001.zip › Figure S2.pdf]

insects

MDPI

Supplementary Materials

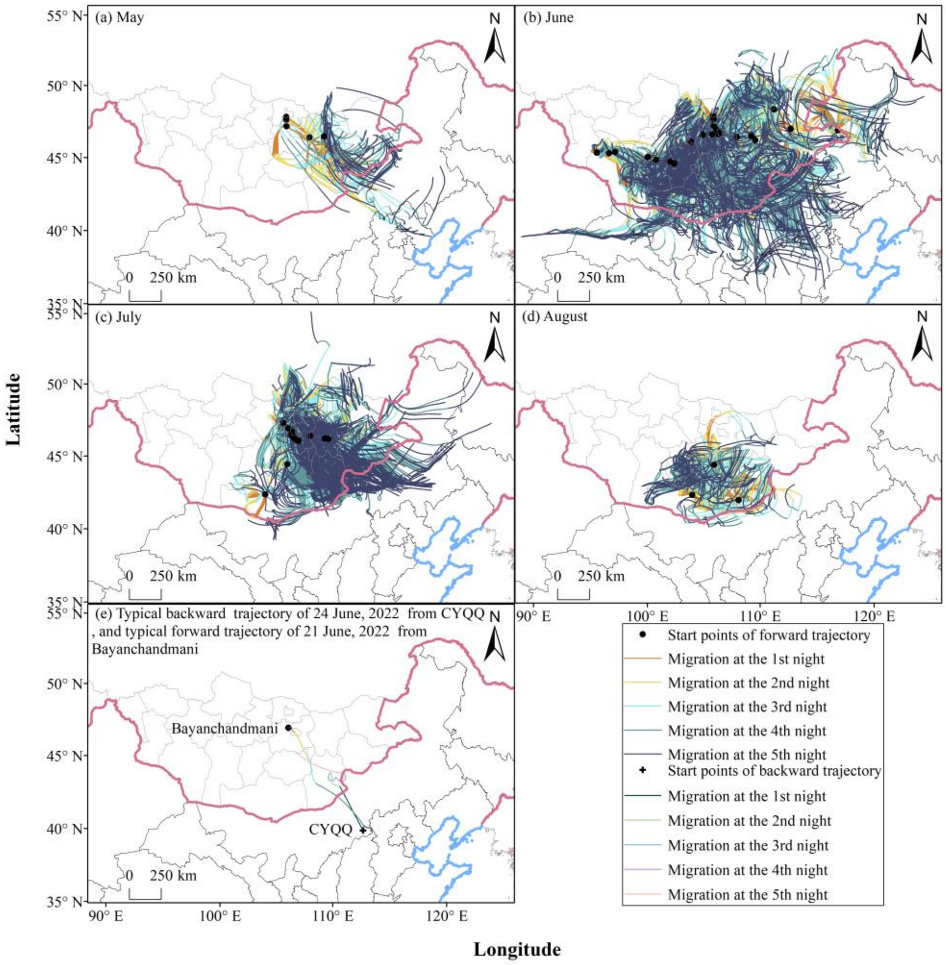

Figure S2. The forward flight trajectories of migratory *L. sticticalis* moths from June to August in Mongolia, 2022.
