# Supplementary material for: Aerial Trajectories and Meteorological Drivers of Transboundary Loxostege sticticalis Migration Across Northern China and Mongolia, 2022
Source: Insects. 2026 Feb 19;17(2):218. doi: 10.3390/insects17020218 (PMC12941310; doi:10.3390/insects17020218)
Supplement: Supplementary file 1 [file insects-17-00218-s001.zip › Figure S3.pdf]

insects

MDPI

Supplementary Materials

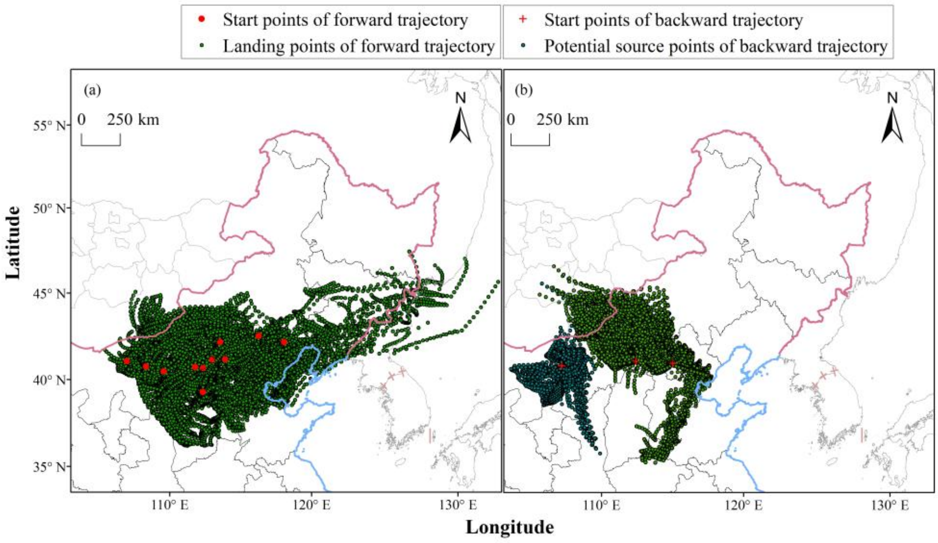

Figure S3. Untrimmed plots of all possible results: forward (a) and backward (b) trajectory point plots of overwintering *L. sticticalis* during the peak period of light-trap catches in northern China, 2022.

 https://doi.org/10.3390/insects17020218
