# Supplementary material for: Aerial Trajectories and Meteorological Drivers of Transboundary Loxostege sticticalis Migration Across Northern China and Mongolia, 2022
Source: Insects. 2026 Feb 19;17(2):218. doi: 10.3390/insects17020218 (PMC12941310; doi:10.3390/insects17020218)
Supplement: Supplementary file 1 [file insects-17-00218-s001.zip › Figure S4.pdf]

Supplementary Materials

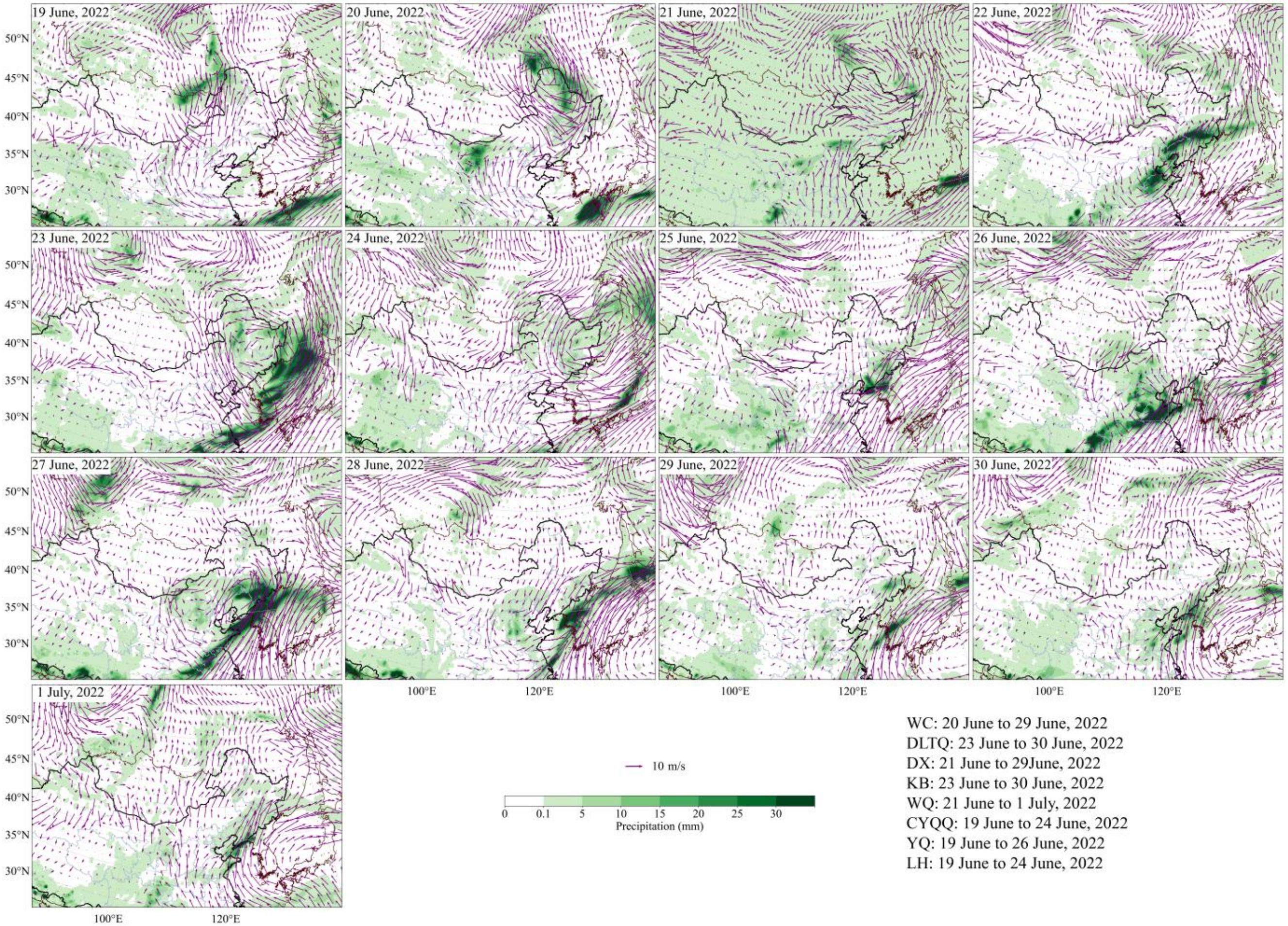

Figure S4. Daily average nighttime wind field at 850 hPa and accumulated precipitation of *L. sticticalis* at representative stations during the dates of peak light-trap catches, 2022.

https://doi.org/10.3390/insects17020218
