# Supplementary material for: Aerial Trajectories and Meteorological Drivers of Transboundary Loxostege sticticalis Migration Across Northern China and Mongolia, 2022
Source: Insects. 2026 Feb 19;17(2):218. doi: 10.3390/insects17020218 (PMC12941310; doi:10.3390/insects17020218)
Supplement: Supplementary file 1 [file insects-17-00218-s001.zip › Figure S5.pdf]

Supplementary Materials

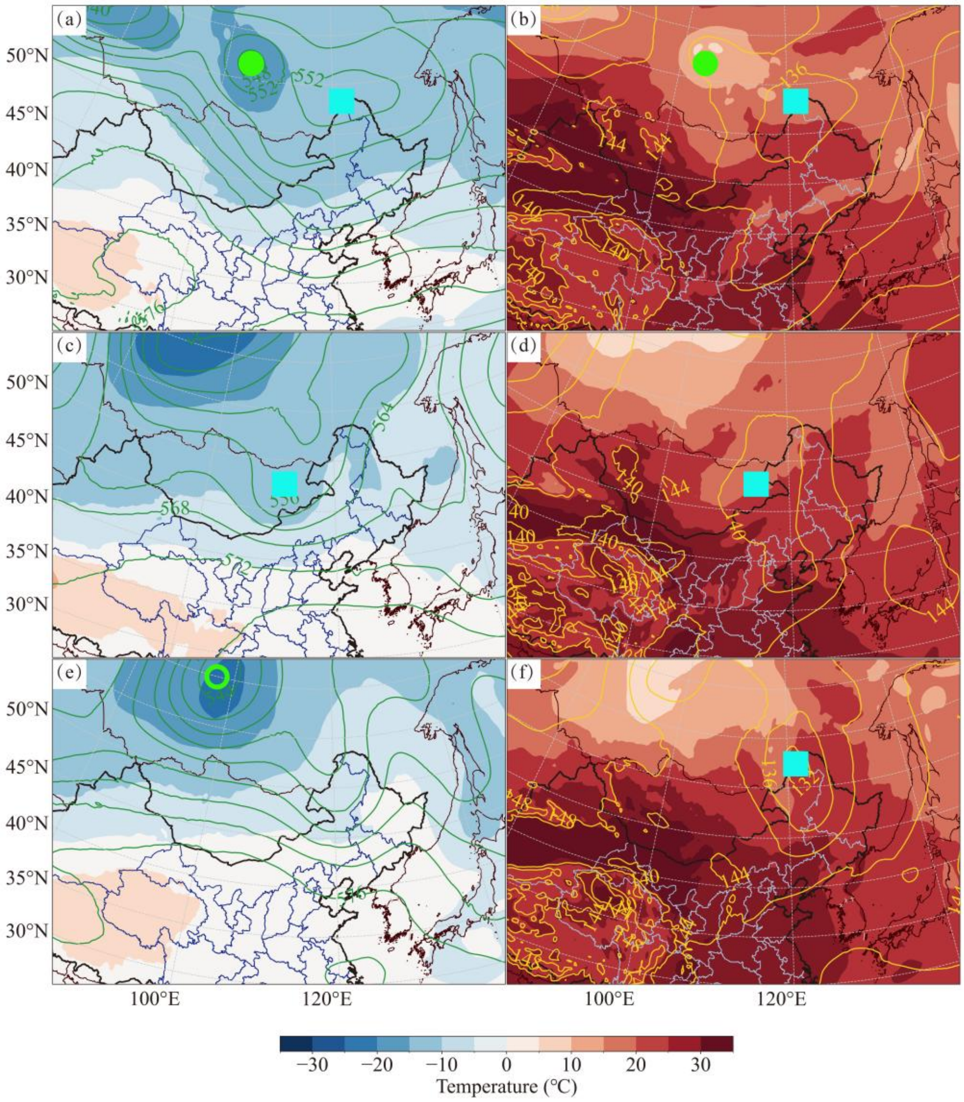

Figure S5. Average nighttime temperature and geopotential height at 500 hPa (**a,c,e**) and 850 hPa (**b,d,f**) during the typical period of the NCCV affecting the Mongolian Cyclone from May to August in 2022. a and b are before the cold vortex absorbed the Mongolian Cyclone on 22 June; (**c,d**) are the Mongolian Cyclone spawned by the trough behind the NCCV on 12 July; (**e,f**) are troughs before the cold vortex, giving rise to a cyclone on 4 August. Cyclone centers were annotated on the figures, with solid circles for the mature NCCV, solid squares for the Mongolian Cyclone, and hollow circles for the cold center.

 https://doi.org/10.3390/insects17020218
