# Supplementary material for: Aerial Trajectories and Meteorological Drivers of Transboundary Loxostege sticticalis Migration Across Northern China and Mongolia, 2022
Source: Insects. 2026 Feb 19;17(2):218. doi: 10.3390/insects17020218 (PMC12941310; doi:10.3390/insects17020218)
Supplement: Supplementary file 1 [file insects-17-00218-s001.zip › Table S1.pdf]

insects

MDPI

Supplementary Materials

Table S1. Possible valid source areas of *L. sticticalis* in northern China documented in recent years.

Province	City	Source
Inner Mongolia	Bayannur City	2022 Survey Data
	Ordos City	2022 Survey Data
	Ulanqab City	2022 Survey Data
	Chifeng City	[95]
	Hinggan League	[95]
	Xilingol League	[12]
	Baotou City	[14]
	Tongliao City	[14]
	Hulun Buir	2024Survey Data
Hebei	Zhangjiakou City	2022 Survey Data
	Chengde City	2022 Survey Data
Beijing	Beijing City	2022 Survey Data
Jilin	Songyuan City	[95]
	Baicheng City	[14]
	Jilin City	2023 Survey Data
Liaoning	Chaoyang City	2022 Survey Data
Heilongjiang	Harbin City	2023 Survey Data
Shanxi	Xinzhou City	2022 Survey Data
	Datong City	[95]
	Shuozhou City	[87]
Shaanxi	Yulin City	[95]
Ningxia	Shizuishan City	[95]
Xinjiang	Altay Prefecture	2023 Survey Data

 https://doi.org/10.3390/insects17020218
