# Supplementary material for: Aerial Trajectories and Meteorological Drivers of Transboundary Loxostege sticticalis Migration Across Northern China and Mongolia, 2022
Source: Insects. 2026 Feb 19;17(2):218. doi: 10.3390/insects17020218 (PMC12941310; doi:10.3390/insects17020218)
Supplement: Supplementary file 1 [file insects-17-00218-s001.zip › Table S2.pdf]

insects

MDPI

Supplementary Materials

Table S2. Probabilities of endpoints of forward trajectory of overwintering *L. sticticalis* in China at the peak of emergence, 2022. Note: N.Korea—The Democratic People's Republic of Korea; HL—Heilongjiang; JL—Jilin; LN—Liaoning; NM—Inner Mongolia; BJ—Beijing; TJ—Tianjin; HE—Hebei; SD—Shandong; SX—Shanxi; SN—Shaanxi.

Province	Start point	Endpoint probability/%													
		Night	Russia	Mongolia	NM	HE	BJ	TJ	SD	SX	SN	LN	JL	HL	N.Korea
		1					23.81		1.02	75.17					
		2				3.67	56	2	1.67	36.67					
SX	DX	3			0.36	6.93	49.64	4.38	2.55	32.48		2.92			0.73
		4			3.4	8.09	40.43	0.85	0.43	31.49		10.64	4.68		
		5			12.79	10.05	26.48	1.37		33.33		6.85	9.13		
		1			33.75	1.25	65								
		2		2.5	50	6.25	41.25								
HE	KB	3		16.67	46.58	7.26	29.49								
		4		32.05	53.85	2.14	11.97								
		5		38.16	61.84										
		1			49.44	0.56	8.33			41.67					
		2			27.22	10	20			42.22		0.56			
NM	LC	3		1.15	51.15		16.09			31.03		0.57			
		4		3.45	60.34		5.75			20.11	10.34				
		5		5.36	61.9		4.17			17.86	10.71				
		1			22.7		26.99					50.31			
		2			14.17	2.5	83.33								
LN	JP	3			35.09	7.89	43.86	13.16							
		4			34.51	3.54	31.86	26.55		3.54					
		5			38.1	7.62	37.14	8.57		8.57					
		1			24.86	70.34						4.8			
		2			34.23	34.83						30.63			0.3
HE	WC	3	0.33		48.52	10.49						27.87	9.84		2.95
		4	1.11	1.11	23.7	26.3						23.7	15.19		8.89
		5	3.8	11.39	13.92	18.99	5.91	2.11				8.86	21.52	2.95	10.55
		1			6.84	14.1	74.5	1.71	2.85						
		2		1.5	24	13.83	51	6.17	2.17			1.33			
HE	WQ	3		6.54	45.42	5.42	27.29	3.18	2.62	1.87		6.73	0.19		0.75
		4		12.53	64.46		9.79	2.05	0.46	0.46		10.25			
		5		23.42	56.67	0.94	2.58	1.17				13.82	0.94		0.47
		1			18.67	2.33	29			50					
		2			16.67	9.33	57.33	0.67	1	14.67		0.33			
NM	FZ	3		1.42	30.5	13.12	33.69	1.77	1.42	15.25		2.84			
		4		3.28	38.69	6.57	21.17	1.09		16.06	1.82	11.31			
		5		3.01	44.36	1.88	25.56			9.4	6.77	7.52	1.5		
		1			25.99	0.56	55.93			17.51					
		2			48.56	7.18	41.09	2.59		0.57					
NM	XH	3		3.4	47.22	9.88	35.19	2.78				1.54			
		4			48.24	3.19	35.78	1.28				11.5			
		5		4.89	33.88	4.89	30.94	1.95				23.13			0.33
NM	DLTQ	1			88.33						11.67				
		2			68.33					23.75	7.92				

		3		60.26			39.74		
		4		56.84			43.16		
		5		51.75		7.02	41.23		
		1		99.72				0.28	
		2		70.83			7.5	21.67	
NM	WLTQQ	3	0.56	47.46		1.13	36.16	14.69	
		4		40.4		10.73	44.35	4.52	
		5		20.4	7.18	10.06	44.83	17.24	0.29
		1		100					
		2	5	89.58				5.42	
NM	WLTZQ	3	1.67	73.75			19.58	5	
		4		72.65			23.08	4.27	
		5		41.45		0.43	34.19	23.93	
