# Supplementary material for: Aerial Trajectories and Meteorological Drivers of Transboundary Loxostege sticticalis Migration Across Northern China and Mongolia, 2022
Source: Insects. 2026 Feb 19;17(2):218. doi: 10.3390/insects17020218 (PMC12941310; doi:10.3390/insects17020218)
Supplement: Supplementary file 1 [file insects-17-00218-s001.zip › Table S3.pdf]

insects

MDPI

Supplementary Materials

Table S3. Probabilities of endpoints of backward trajectory of overwintering *L. sticticalis* in China at the peak of emergence, 2022. Note: NM—Inner Mongolia; BJ—Beijing; TJ—Tianjin; HE—Hebei; SX—Shanxi; SN—Shaanxi; NX—Ningxia.

**Province**	**Start point**	**Endpoint probability/%**								
		**Night**	**Mongolia**	**NM**	**HE**	**BJ**	**TJ**	**SX**	**SN**	**NX**
NM	CYQQ	1	6.67	93.33						
		2	25	75						
		3	14.17	85.83						
		4	15.83	84.17						
		5	29.63	70.37						
NM	LH	1		100						
		2		94.92					5.08	
		3	3.45	96.55						
		4	0.86	99.14						
		5	0.97	94.17						4.85
BJ	YQ	1		17.94	39.01	43.05				
		2	3.59	34.08	47.98	14.35				
		3	2.69	54.71	26.01	14.8	1.79			
		4	4.05	57.66	38.29					
		5	6.02	49.54	40.28	0.46		3.7		
