# Supplementary material for: Aerial Trajectories and Meteorological Drivers of Transboundary Loxostege sticticalis Migration Across Northern China and Mongolia, 2022
Source: Insects. 2026 Feb 19;17(2):218. doi: 10.3390/insects17020218 (PMC12941310; doi:10.3390/insects17020218)
Supplement: Supplementary file 1 [file insects-17-00218-s001.zip › Table S4.pdf]

insects

MDPI

Supplementary Materials

Table S4. Probabilities of endpoints of forward trajectory of *L. sticticalis* from Mongolia during May to August, 2022. Note: HL—Heilongjiang; JL—Jilin; LN—Liaoning; NM—Inner Mongolia; BJ—Beijing; HE—Hebei; SD—Shandong; SX—Shanxi; SN—Shaanxi; GS—Gansu; XJ—Xinjiang.

Monitoring Time	Night	Endpoint probability (Actual monitoring date / other date of MTW / total date of MTW)/%												
		Russia	Mongolia	NM	XJ	GS	SN	SX	HE	BJ	SD	LN	JL	HL
May	1		-/100/100											
	2	-/0.16/0.16	-/84.42/84.42	-/15.11/15.11					-/0.31/0.31					
	3	-/1.39/1.39	-/68.62/68.62	-/23.8/23.8					-/6.18/6.18					
	4	-/5.98/5.98	-/62.4262.42	-/20.86/20.86					-/7.21/7.21	-/3.07/3.07		-/0.46/0.46		
	5	-/4.08/4.08	-/50.3/50.3	-/36.1/36.1					-/8.16/8.16	-/0.91/0.91		-/0.45/0.45		
June	1	1.62/5.06/4.69	94.64/91.67/91.98	3.74/3.27/3.32										
	2	8.29/7.53/7.66	82.74/86.14/85.71	8.97/6.26/6.57		-/0.08/0.07								
	3	9.46/9.76/9.74	68.84/77.6/76.57	20.85/11.06/12.19		-/0.63/0.56	-/0.57/0.51	0.17/0.07/0.08	0.67/0.29/0.33				-/0.02/0.02	
	4	9.4/9.54/9.53	66.67/68.7/68.47	17.95/17.72/17.73	-/0.21/0.19	-/1.49/1.32	-/0.71/0.63	2.39/0.35/0.59	3.25/0.86/1.13	0.26/0.02/0.05			0.09/0.21/0.2	-/0.2/0.18
	5	8.5/7.04/7.25	68.71/65.69/66.01	14.37/19.5/18.9	-/0.74/0.66	-/2.03/1.8	-/0.74/0.66	1.79/0.88/0.99	4.34/2.03/2.3	1.62/0.09/0.26	-/0.01/0.01	-/0.11/0.1	-/0.28/0.24	0.68/0.85/0.83
July	1	1.42/1.72/1.75	98.58/98.22/98.19	-/0.06/0.06										
	2	4.15/7.87/7.6	95.85/91.98/92.27	-/0.15/0.13										
	3	2.95/8.06/7.59	97.05/89.07/89.85	-/2.87/2.56										
	4	2.89/5.18/4.99	76.62/86.56/85.41	20.49/8.27/9.61										
	5	2.83/4.98/4.74	45.75/77.67/74.08	50.25/17.07/20.8									1.17/0.03/0.16	-/0.25/0.22
August	1		100/99.73/99.77	-/0.27/0.23										
	2	-/0.09/0.08	88.33/94.92/94.02	11.67/4.99/5.9										
	3	-/0.09/0.08	84.48/94.59/93.24	15.53/5.32/6.69										
	4		83.33/95.63/93.93	16.67/4.376.07										
	5		87.36/96.54/95.31	12.64/3.46/4.69										

https://doi.org/10.3390/insects17020218
