# Supplementary material for: Aerial Trajectories and Meteorological Drivers of Transboundary Loxostege sticticalis Migration Across Northern China and Mongolia, 2022
Source: Insects. 2026 Feb 19;17(2):218. doi: 10.3390/insects17020218 (PMC12941310; doi:10.3390/insects17020218)
Supplement: Supplementary file 1 [file insects-17-00218-s001.zip › Table S5.pdf]

Supplementary Materials

Table S5. Habitat suitability in the core areas of Mongolia in 2022.

Monitoring Location	Mean Temperature at 2 m Above Ground/°C	January Average Temperature at 2 m Above Ground/°C	Annual Precipitation/mm	Precipitation During the Window Period/mm
6 km to the south from Tsenkhermandal soum	0.2	-18.7	325.4	11.1
Baganuur, Kherlen river	-1.2	-19.6	340.6	3.5
Baruunharaa soum	1.6	-16.8	312.5	19.8
Khustain davaa	2.1	-16.1	298.3	22.4
Dugan khad	0.4	-17.6	392.2	29.1
Bayanuul soum, Uikhan	0.2	-19.3	472.7	36.8
Elsen tasarkhai	1.3	-17.4	284.4	28.5
Khairkhandulaan soum	1.8	-15.7	135.8	4.6
Nariin teel soum	-0.1	-17.3	146.8	2.9
Bulgan soum	1.9	-17.9	330.2	31.3
Site1	3	-15.9	82	1.6
Bombogor soum, Baidragiin guur	2	-16.9	78.3	1.5
Khalkh gol soum, Tultiin hyasaa	0.5	-22.2	340.8	25.2
Site2	2.7	-14.1	69.9	0.2
on the way to Bayannuur	0.8	-21.4	283.6	16.6
Altanbulag soum, on the way to Khustai	0.1	-20.2	307.8	21.2
Sharga soum	6.9	-14.5	22.8	0
Tonkhil soum	0.5	-18.4	93.8	1
Bayanchandmani sum	-0.5	-18.4	437.8	16.7
River Kherlen	-1.2	-19.6	340.6	10.7
Moron soum	0.8	-19.1	267.3	50.6
Nearby Ulaanbaatar1	-0.9	-19.5	331.8	16.4
Nearby Ulaanbaatar2	-0.9	-19.5	331.8	16.4
Nearby Ulaanbaatar3	-0.9	-19.5	331.8	16.4
Nearby Ulaanbaatar4	-0.7	-19	308	13.5
Nearby Ulaanbaatar5	-0.7	-19	308	13
Nearby Ulaanbaatar6	-0.7	-19	308	13
Nearby Ulaanbaatar7	-0.7	-19	308	13
Nearby Ulaanbaatar8	-1	-19.3	330.7	16.7
Nearby Ulaanbaatar9	-1	-19.3	330.7	16.7
Nearby Ulaanbaatar10	-1.2	-19.4	354.8	17.8
Nearby Ulaanbaatar11	-1.2	-19.4	354.8	17.2
Nearby Ulaanbaatar12	-1.1	-19	340.5	16.8
Nearby Ulaanbaatar13	-1.1	-19	340.5	16.8
Nearby Ulaanbaatar14	-1.1	-19	340.5	16.8
Nearby Ulaanbaatar15	-0.6	-19	312.8	13.9

on the way to Chingis city (Poa sp.)1	0.7	-18.6	284.9	29.8
on the way to Chingis city (Raps)2	0.7	-18.6	284.9	29.8
Jargalant1	0.9	-17.8	331.6	24.3
Jargalant2	-0.5	-18.4	437.8	34.8
Site3	2.7	-15.8	163.4	2
Tsogtsetsii	3.8	-12.8	108.1	5.2
on the way to Khatanbu-lag2	7.6	-12.1	78.3	6.5
